# Causes of Decay of Teeth

**Published:** 1874-07

**Authors:** 


### ARTICLE VII.

### *The Causes of Decay of Teeth.*

It has been charged against our brethren of the dental specialty that they are wofully at fault in regard to knowledge of the commonest of all things—caries of the teeth That they extract teeth with skill, and stop them with even more skill, and in a nobly conservative spirit is admitted; but the causes of decay in the teeth have remained obscure.

The investigations of Leber and Rottenstein into this subject have at least the charm of pointing to definite conclusions. We have read their work, in the form of an American translation by Dr. Thomas H. Chandler, D. M. D., Professor of Mechanical Dentistry in the Dental School of Harvard University, and published by Trubner. They admit, of course, that there are differences of teeth, constitutional and connected with race, making teeth more or less resistant to the great influences which determine decay. These are not, according to these authors, internal or vital so much as external and chemical. The process of decay begins from the surface, and if it can be controlled or arrested at the surface it is entirely controlled.

The great causes of caries are two—viz., acids and a certain fungus found abundantly in the mouth, the *Leptothrix buccalis.* This latter agent is characterised by certain microscopic appearances, and by its reaction with iodine and acids, which give to the elements of leptothrix a beautiful violet tinge. Under the microscope, the fungus appears as a grey, finely granular mass or matrix, with filaments delicate and stiff, which erect themselves above the surface of this granular substance so as to resemble an uneven turf. The fungus attains its greatest size in the interstices of the teeth.

No one can deny, nowadays, the action of acids on the teeth—even weak acids—in dissolving the salts of the enamel and the dentine. All acids, both mineral and vegetable, act promptly on the teeth. Various experiments as to the action of acids on dental tissues are given, making the enamel, naturally transparent, first white, opaque, and milky, and, in a more advanced state, chalky, and then the dentine more transparent, and softer, so as to be cut with a knife. The acids which may naturally effect the first changes in the production of caries are such as are taken with food, or in medicines, or such as are formed in the mouth itself by some abnormality in our secretions, which should be alkaline, or by an acid fermentation of particles of food.

But acids alone will not account for all the phenomena of caries in the teeth. They play a primary and principal part, making the teeth porous and soft. In this state, the tissues having lost their normal consistency, fungi penetrate both the canaliculi of the enamel and of the dentine, and by their proliferation, produce softening and destructive effects much more rapid than the action of acids alone is able to accomplish. It is not pleasant to think that fungi exists in the mouths of all but the very cleanest people. Bowditch, in examining forty persons of different professions, and living different kinds of life, found, in almost all, vegetable and animal parasites. The parasites were numerous in proportion to the neglect of cleanliness. The means ordinarily employed to clean the teeth had no effect on the parasites, whilst soapy water appeared to destroy them.

If this be a true version of the causes of caries—the action of acids, supplemented by the action of fungi—then it follows that the great means of preserving teeth is to preserve the most scrupulous cleanliness of the mouth and teeth, and to give to the rinsing liquids a slightly alkaline character, which is done by the admixture of a little soap. This is not so pleasant a dentrifice as some, but it is effective and scientific.

Acids not only dissolve the salts of the teeth, but favor the increase of the fungi of the mouth. No increase of fungi and no action on the dental tissues occurs in solutions slightly alkaline, such as a weak solution of soap

The good effects of stopping teeth, in the light of these experiments, are intelligible. The penetration of acids and fungi is prevented.—*London Lancet.*

